# Nonrigid 3D Medical Image Registration and Fusion Based on Deformable Models

**DOI:** 10.1155/2013/902470

**Published:** 2013-04-18

**Authors:** Peng Liu, Benjamin Eberhardt, Christian Wybranski, Jens Ricke, Lutz Lüdemann

**Affiliations:** ^1^Department of Radiotherapy, Universitätsklinikum Essen, Hufelandstraße 55, 45147 Essen, Germany; ^2^Department for Radiology and Nuclear Medicine, Universitätsklinikum Magdeburg, Leipziger Straße 44, 39120 Magdeburg, Germany

## Abstract

For coregistration of medical images, rigid methods often fail to provide enough freedom, while reliable elastic methods are available clinically for special applications only. The number of degrees of freedom of elastic models must be reduced for use in the clinical setting to archive a reliable result. We propose a novel geometry-based method of nonrigid 3D medical image registration and fusion. The proposed method uses a 3D surface-based deformable model as guidance. In our twofold approach, the deformable mesh from one of the images is first applied to the boundary of the object to be registered. Thereafter, the non-rigid volume deformation vector field needed for registration and fusion inside of the region of interest (ROI) described by the active surface is inferred from the displacement of the surface mesh points. The method was validated using clinical images of a quasirigid organ (kidney) and of an elastic organ (liver). The reduction in standard deviation of the image intensity difference between reference image and model was used as a measure of performance. Landmarks placed at vessel bifurcations in the liver were used as a gold standard for evaluating registration results for the elastic liver. Our registration method was compared with affine registration using mutual information applied to the quasi-rigid kidney. The new method achieved 15.11% better quality with a high confidence level of 99% for rigid registration. However, when applied to the quasi-elastic liver, the method has an averaged landmark dislocation of 4.32 mm. In contrast, affine registration of extracted livers yields a significantly (*P* = 0.000001) smaller dislocation of 3.26 mm. In conclusion, our validation shows that the novel approach is applicable in cases where internal deformation is not crucial, but it has limitations in cases where internal displacement must also be taken into account.

## 1. Introduction

 In many clinical tasks it is necessary to acquire images using different modalities such as magnetic resonance imaging (MRI), positron emission tomography (PET), and computed tomography (CT), which often provide complementary information on anatomy and tissue function. Combination of these multimodal images can improve the diagnosis by providing synergistic information. Image registration is an important tool for fusion of medical images. It generates an image that simultaneously displays the information of the reference and the registered image. Image registration aims at identifying corresponding points in two images using spatial transform. Due to the spatial difference in the local coordinate systems of images acquired with different imaging modalities registration has to align the images.

Another application of image registration is serial imaging of a patient using the same imaging modality, which is often required for purposes such as treatment planning and monitoring [1], evaluation of disease development [2, 3], and tracking of contrast bolus propagation in perfusion studies [4–6]. Registration is required to correct for the motion caused by patient movement and respiration and to compensate for the displacement of structures resulting from different patient positions in serial imaging studies. Although many spatial displacements can be traced back to rigid movement and are easy to correct, elastic deformation is required to describe the movement of many anatomic structures such as the liver. Thus nonrigid registration is often required for both repeated acquisitions using the same image modality and examinations using different image modalities.

Rigid transformation, which allows translation and rotation, and affine registration, which allows shearing and scaling, are widely used for image fusion in a clinical setting. However, since the human body is intrinsically deformable, rigid techniques often provide insufficient registration. Thus elastic or nonrigid methods are required to cope with local differences between images. While the number of parameters is limited to six for rigid transformation and to twelve for affine transformation, nonparametric elastic transformation requires a transformation vector for each voxel; that is, the number of parameters is three times that of image voxels. The huge number of parameters generates two basic problems for elastic transformation. First, the computing time is enormous. Second, the intrinsic image information is in general not sufficient to exactly and independently estimate the transformation vector of each voxel.

Therefore, elastic image registration uses models to limit the number of parameters. However, one can also benefit from the fact that the number of effective transformation parameters is actually much smaller. It is obvious that a tissue voxel in general cannot move independently of a neighboring voxel. Thus elastic registrations exploit the fact that the transformation field should be smooth [7, 8]. A smooth transformation field adequately describes the actual dislocation of most voxels. On the other hand, in some instances, the entire inner organ moves, while movement of organ voxels relative to each other is small. It was demonstrated that movement of organs such as the prostate [4], kidney [6], or liver [1] can be estimated, in a first approximation, by a rigid or affine transformation. In consequence, the transformation field for some inner organs is not smooth. The difference between the transformation vectors inside an organ is small, while the difference between the transformation vectors at the organ surface might be much larger.

Most approaches to elastic registration are based on matching signal intensities [9–12], which is limited to special applications [10, 11] or make use only of a smooth transformation field [7–9, 13, 14]. In our paper we introduce a novel geometry-based three-dimensional nonrigid registration approach. The method is based on the assumption that organ movement is primarily effective at the organ surface. We assume a smooth transformation field inside an organ and free transformation at the organ surface. Thus, our registration algorithm is a geometrical method based on segmentation. This kind of registration requires preliminary segmentation of the anatomical structure of interest on the reference or model image. Binary structures such as contours and surfaces can be generated by labeling them manually or (semi)automatically using an advanced algorithm. The basic idea is to use deformable models to guide image registration. With such models, nonrigid volume deformations are inferred from surface deformations. The transformation required for registration is then calculated by minimizing the distance of the contour points. Furthermore, a fusion technique based on the inferred volume deformation is introduced. Factors which may influence the performance of the method are discussed.

## 2. Theory

### 2.1. Concept

 Our registration algorithm is a geometrical method based on segmentation. This kind of registration requires segmentation of the anatomical structure of interest on the reference or model image to generate a binary structure. Segmentation can be performed manually or (semi)automatically using an advanced algorithm. An active surface mesh is generated from the segmentation of the reference images. Since an active surface mesh is a 3D deformable model which extends active contours [15] to 3D and thus can be adapted to be applied to the edges of an edge map extracted from the complementary image of the registration or fusion, we believe that mesh displacement could indicate movement of the organ. In our approach, surface evolution in the edge map leads to surface deformations described by the displacement of surface mesh points. The resulting nonrigid volume deformation vector field for registration or fusion inside the region of interest (ROI) is predicted by solving the reverse problem of free-form deformation (FFD) [16]. FFD allows to obtain the first experimental result of prediction based only on geometrical knowledge.

#### 2.1.1. Active Surface

 Deformable models can be applied to image edges [17] with large image intensity gradients by using an optimization method. In our approach we use a 3D extension of active contours called active surfaces. While the level set method uses implicit surfaces [18, 19], we use explicit surfaces constructed from tetrahedres.

The basic concept of active models in general, and of active surfaces in particular, is to give the models physical characteristics in terms of energy. Internal energy and external energy are two widely used concepts in this context: internal energy *E*_int⁡_ describes the internal deformation characteristics of the model, that is, the smoothness of the surface. External energy *E*_ext_ describes the environmental influence on the model. Here “environment” refers to images or their filtered forms, on which the models are settled and transformed. Basically, external energy is extracted from features such as object boundaries, where the image gradient has its local maximum and is often described as potential energy.

Based on the definition of energy, the segmentation process ideally minimizes the total energy, *E*_total_, of the model:

(1) $$\begin{array}{c} E_{\text{total}}=E_{\mathrm{int}}+E_{\text{ext}}. \end{array}$$

As a result the active model will be attracted to the object boundary, where total energy is the lowest. To achieve this desired property of the model, the shape of the object of interest is supposed to be regular and smooth so that, at the object boundary, the bending energy, defined by rigid force ${\vec{F}}_{\text{rigid}}$, and the stretching energy, defined by elastic force ${\vec{F}}_{\text{elastic}}$, compensate for the potential energy that defines the external energy. This can also be seen as a state of force balancing of the internal forces (${\vec{F}}_{\text{rigid}}$, ${\vec{F}}_{\text{elastic}}$) and the external force (${\vec{F}}_{\text{ext}}$):

(2) $$\begin{array}{c} {\vec{F}}_{\text{total}}={\vec{F}}_{\text{rigid}}+{\vec{F}}_{\text{elastic}}+{\vec{F}}_{\text{ext}}=0. \end{array}$$

Since the active surface moves during the energy-minimizing procedure, we thus describe the whole process as a surface evolution function of time:

(3) $$\begin{array}{c} \frac{\partial S\left(s,t\right)}{\partial t}=\tau \left(t\right){\vec{F}}_{\text{total}}\left(t\right), \end{array}$$

where *S* is the active surface defined as a set of surface mesh points *s* at time *t*. Vector $\tau (t){\vec{F}}_{\text{total}}(t)$ refers to the increment of surface movement at time *t*, where the time-dependent scale factor *τ* is used to control movement speed, ensuring numerical stability under the *Courant-Friedrichs-Lewy* (CFL) condition [20]. The steady state, in which total energy is at its minimum, is reached if the increment for optimization approximates zero.

#### 2.1.2. Free-Form Deformation

 Our method uses FFD to describe deformation of the object of interest, which is embedded in a control grid with a given resolution in three dimensions of (*l* + 1)(*m* + 1)(*n* + 1). Using its local coordinate system defined by unit vectors $\vec{S}$, $\vec{T}$, and $\vec{U}$, a grid point ${\vec{p}}_{ijk}$ of the FFD can be defined as

(4) p→ijk=x→0+ilS→+jmT→+knU→(i∈[0,l],j∈[0,m],k∈[0,n]),

where ${\vec{x}}_{0}$, described by its global coordinates, is the origin of the local coordinate system. Using trivariate tensor product Bernstein polynomial, the position in the global coordinate system of any point $\vec{x}$ inside the FFD grid can be interpolated via

(5) x→=∑i=0 l∑j=0 m∑k=0nCliCmjCnk(1−χ)l−iχi(1−ψ)m−jψj·(1−ω)n−kωkp→ijk,

where *C*_*l*_^*i*^, *C*_*m*_^*j*^, and *C*_*n*_^*k*^ are the binomial coefficients in respect of  *l*, *m*, and *n*. Furthermore *χ*, *ψ*, and *ω* are the local coordinates of $\vec{x}$, where

(6) χ=T→×U→·(x→−x→0)T→×U→·S→,ψ=S→×U→·(x→−x→0)S→×U→·T→,ω=S→×T→·(x→−x→0)S→×T→·U→.

Since all surface mesh points are settled inside the FFD grid, a surface point $\vec{s}$ can be interpolated as well using the above interpolation function (5) by replacing $\vec{x}$ with $\vec{s}$. Since we always take a set of points, in our case the active surface, into consideration, we use the matrix description of (5) for the whole surface **S**:

(7) $$\begin{array}{c} S=B\cdot P, \end{array}$$

where **S** is an *N* × 3 matrix, **B** is an *N* × (*l* + 1)(*m* + 1)(*n* + 1) matrix, and **P** is an (*l* + 1)(*m* + 1)(*n* + 1) × 3 matrix for a given number *N* of surface mesh points considered inside the FFD grid. Since **B** solely describes the deformation for a given set of grid control points **P**, it is normally referred to as deformation matrix. Based on the above matrix description (7) the following applies:

(8) $$\begin{array}{c} S^{*}=B\cdot P^{*}, \end{array}$$

where P* describes the set of displaced control points and S* refers to the deformed surface resulting from the right side of the function, given that **B** is unchanged. This means, if we know the displacement of **P**, we can calculate the deformation of **S**. But note that our goal is to reversely solve the problem; that is, we have a set of deformed surface mesh points S* and wish to calculate P* in order to further use P* for interpolating the deformation of any point $\vec{x}$ of the volume inside the surface as described in (5). Since the number of surface mesh points is much larger than that represented in the FFD control grid, using the pseudoinverse **B** ^+^ of **B** to solve

(9) $$\begin{array}{c} P^{*}=B^{+}S^{*} \end{array}$$

will provide the solution to an overdetermined system rather than giving us a sufficient solution to the reverse problem in general. In our approach, we therefore solve the problem by using the Levenberg-Marquardt algorithm [21, 22], a method of least squares, in order to minimize the squared distance between **S** and S*:

(10) $$\begin{array}{c} P^{*}=\arg\underset{P}{\min}{||S-S^{*}||}^{2}. \end{array}$$

## 3. Material and Methods

### 3.1. Numerical Implementation

 In our approach we use an active surface 3D deformable model to guide the registration. The active surface is generated through triangulation from a preliminary segmentation of the object of interest on the model image and later adapted to the edges of the reference image as a deformable model by defining the internal and external forces acting on it. The elastic force acting on a surface mesh point is the sum of tensile forces from its neighboring points. The rigid force is described as a linear prediction of the tensile forces from its neighboring points and their neighbors [23]. The active surface is optimized by applying the finite difference method (FDM) to an *inverse edge map* [24] of the reference image.

After minimizing the total energy a deformed version of the original surface is displayed at the boundary of the object of interest on the reference image. Thus we have a one-to-one mapping of the surface mesh points as well. Based on the mapping an FFD control grid wrapping the original active surface can be deformed by solving the inverse problem of the FFD. We then use the computed deformation of the FFD control grid, along with the deformation matrix of the FFD, to transform the voxels inside the original surface of the model image onto the reference image; hence image information such as intensity saved in the object of interest of the model image can be transferred into the deformed object described by the deformed surface on the reference image, which fulfills the registration task.

Conversely the procedure can also begin with a preliminary segmentation on the reference image in order to adapt the initial surface mesh to the reference image on the model image, thereby transferring the volume deformation from the reference to the model image. Using the aforementioned FFD interpolation method, image intensity in the deformed FFD on the model image can be sampled and traced back to the original object of interest in the reference image. In this way, fusion is accomplished.

Our method has been implemented as AMIRA (http://www.amira.com) modules. AMIRA is an advanced 3D visualization software developed by Konrad-Zuse-Zentrum für Informationstechnik, Berlin (http://www.zib.de/de/home.html) and distributed by Visage Imaging, Berlin (http://www.visageimaging.com). AMIRA is highly modularized using C++ to offer visualization and image analysis pipelines based on modules. Since our method is a twofold approach consisting of segmentation and subsequent FFD computation, the AMIRA modules are implemented in two packages, *hxactcontour* and *hxffd*. Furthermore, an upper level package *hxsera* (*sera* stands for segmentation-based elastic registration algorithm.) wraps the two packages for user-friendly access to the entire procedure.

The computational complexity of the segmentation task is linearly dependent on the number of surface mesh points and can be described as *O*(*n*) using big-*O* notation [25]. When performing kidney segmentation, for example, we in general had from 10,000 to 20,000 points to process. In comparison the complexity of FFD computation is *O*(*m* · *n*), where *m* is the number of FFD control points and *n* is the number of surface mesh points. The FFD grid we used typically had a resolution of 5 × 5 × 5 to 10 × 10 × 10. Thus the number of FFD control points is considerably smaller than that of surface mesh points.

### 3.2. Image Data

To test the feasibility of our approach we first applied it to two contrast-enhanced dynamic computed tomography (CT) examinations consisting of a total of 41 3D datasets of the kidneys obtained in two patients who underwent routine clinical evaluation of renal perfusion. Each CT series was acquired using 320 slices with a 512 × 512 voxel in-plane resolution. The CT examinations were performed on a *TOSHIBA Aquilion ONE* with a total acquisition time of 1 min for the complete dataset while the iodine contrast agent was administered as a bolus. To reduce the absorbed dose the tube current was minimized.

The first patient received 90 mL contrast medium and was examined with a CT scanner tube voltage of 120 kV and tube current of 150 mA. 24 3D CT datasets were acquired with a spatial resolution of 0.571 mm × 0.571 mm and a slice thickness of 0.5 mm. The second patient received 120 mL contrast medium and underwent CT scanning with a tube voltage and current of 100 kV and 100 mA, respectively. Seventeen 3D CT datasets were acquired with a spatial resolution of 0.702 mm × 0.702 mm × 0.5 mm. Due to the high signal noise of the low dose scans the effective spatial resolution was lower than the nominal resolution of the scans. Therefore, the 3D CT datasets were resampled to a resolution of 256 × 256 × 160 for the study.

We further used our method for multimodal registration in patients undergoing imaging of the liver, which is a more elastic organ than the kidney. Twenty patients treated by routine clinical brachytherapy [26] were investigated. A 3D CT and a 3D MRI interventional dataset from each patient were acquired no later than 1 hour after brachytherapy catheter positioning. One of the two 3D datasets was used for therapy planning. Furthermore, follow-up MRI performed several months after treatment was available for all patients. Because of the long interval between the intervention and follow-up MRI, there may be considerable liver movement and deformation in the follow-up images compared with the initial planning 3D dataset.

All axial CT scans of the liver were acquired with a resolution of 512 × 512, but the number of slices ranged from 31 to 322, resulting in severe partial volume effects in the CT scans acquired with a lower number of slices. The averaged spatial resolution of the CT scans is 0.743 mm × 0.743 mm × 3.04 mm compared with 1.187 mm × 1.187 mm × 2.50 mm for MRI, where an invariable slice thickness of 2.50 mm applies to all cases. T1-weighted volume-interpolated 3D gradient echo MR images were acquired during catheter positioning on an open bore Philips 1.0 Tesla MR with a more inhomogeneous signal distribution.

The following sequence parameters were used: echo time (TE) 2.14 ms, repetition time (TR) 4.3 ms, echo train length 104, 122 phase-encoding steps, flip angle 12°, image matrix 320 × 320, FOV 360 mm, 58% sampling, 75 slices, slice thickness 5.0 mm, slice spacing 2.5 mm. Additional T1-weighted 2D GRE MR images were acquired 12 weeks after brachytherapy for assessing the response to treatment on a Philips Achieva 1.5 Tesla MR imager using the following sequence parameters: TE 5.0 ms, TR 110 ms, 192 phase-encoding steps, flip angle 7°, image matrix 512 × 512, FOV 430 mm, 75% sampling, 70% phase FOV, 28 slices, slice thickness 8.0 mm, slice spacing 9.0 mm. MR images with incomplete depiction of the liver were excluded from evaluation.

### 3.3. Evaluation Methods

 To validate our approach, we used empirical methods, which are subcategorized into discrepancy and goodness methods as described in [27]. Discrepancy methods depend on an optimal reference, which is generally known as the gold standard and has been verified by experts. Goodness methods do not need a reference but rather depend on some preferable characteristics to describe and thus judge the performance of the algorithm.

In our validation we investigated alignment of the volume and misalignment of contours by using two different kinds of discrepancy features. The *Dice similarity coefficient* (DSC) [28, 29] was used to evaluate volume alignment

(11) $$\begin{array}{c} \text{DSC}=\frac{2|A\cap B|}{\left|A\cup B\right|}, \end{array}$$

where *A* is the volume of the segment to be validated and *B* the volume of the gold standard. For contour misalignment, we evaluated the *Hausdorff distance* [30]

(12) dh(kA,kB)=max⁡(max⁡i{d(ai,kB)},max⁡j{d(bj,kA)})(ai∈kA,bj∈kB)

with

(13) $$\begin{array}{c} d\left(a_{i},k_{B}\right)=\underset{j}{\min}\left|b_{j}-a_{i}\right| \end{array}$$

and the averaged contour misalignment

(14) $$\begin{array}{c} \bar{d}\left(k_{A},k_{B}\right)=\frac{\sum_{i}^{}\min_{j}\left|b_{j}-a_{i}\right|}{\left|k_{A}\right|}, \end{array}$$

where *k*_*A*_ and *k*_*B*_ are sets of contour points of the segment to be validated and the gold standard, respectively, and *a*_*i*_ and *b*_*j*_ represent the contour points of sets *k*_*A*_ and *k*_*B*_. Note that $\bar{d}(k_{A},k_{B})$ and $\bar{d}(k_{B},k_{A})$ are generally not equal [31], so that the combined average of the contour misalignment was used for evaluation.

Furthermore, two different goodness methods were used to validate the segmentation approach: the *intraregion uniformity* [32] and the *gray level contrast* [27]. Intraregion uniformity is based on the assumption that the regions that have been segmented should have a uniform distribution of gray levels, which means that variance within each region should be small. ${\bar{g}}_{j}$ is defined by the signal intensity *g*_*i*_ of voxel *i* in region *R*_*j*_ that has been segmented with mean intensity in *R*_*j*_ as

(15) $$\begin{array}{c} {\bar{g}}_{j}=\sum_{i\in R_{j}}\frac{g_{i}}{V_{j}}, \end{array}$$

where *V*_*j*_ describes the volume of *R*_*j*_. The variance of *R*_*j*_ is defined as

(16) $$\begin{array}{c} \sigma_{j}^{2}=\sum_{i\in R_{j}}\frac{g_{i}-{\bar{g}}_{j}}{V_{j}}. \end{array}$$

Based on the intensity variance defined previously, uniformity is defined as

(17) $$\begin{array}{c} U=1-\sum_{R_{j}}\frac{V_{j}\sigma_{j}^{2}}{V_{\text{all}}\sigma_{\max}^{2}}, \end{array}$$

where *V*_all_ is the sum of all volumes that have been segmented and *σ*_max⁡_^2^ is a normalization factor defined as

(18) $$\begin{array}{c} \sigma_{\max}^{2}=\frac{{\left(g_{\max}-g_{\min}\right)}^{2}}{2}, \end{array}$$

where *g*_max⁡_ and *g*_min⁡_ are the absolute maximum and minimum of the signal intensities from all segmented regions, respectively. In comparison, the signal contrast takes the intensity difference between the segmented region and its background into consideration and assumes that contrast should be large. For the averaged signal intensity of the segmented region, *f*_0_, and the averaged background signal intensity, *f*_*b*_, signal contrast is defined as

(19) $$\begin{array}{c} \text{GC}=\frac{\left|f_{0}-f_{b}\right|}{f_{0}+f_{b}}. \end{array}$$

Furthermore, to validate our final registration result, we used a goodness method based on our empirical study, assuming that the change in signal intensity should be small within the region of interest (ROI) but large between the ROI and its background. This means that an accurate registration should yield a small variance in intensity, while a poor registration should yield much greater variance. Here we measure the standard deviation of intensity of the registered image within the reference ROI that was segmented by experts as gold standard (see Appendix A).

### 3.4. Evaluation

As mentioned above, we first applied our approach to dynamically acquired renal CT scans because the kidneys are relatively rigid organs. Segmentation and registration were evaluated separately. The two patients are numbered *P*.#1 and *P*.#2, and their 3D datasets are numbered consecutively beginning with the first acquisition of the dynamic series.

To reduce intra- and interexpert variability gold standards were obtained for the discrepancy methods using an iterative *expectation-maximization* method [33, 34] with an expert performing five segmentations for each 3D dataset. To validate the registration result we used our goodness feature—the standard deviation of intensity—to compare our registration with an intensity-based affine registration using mutual information [35] as the optimization metric.

We further validated our method for multimodal registration of the liver, which is more elastic than the kidney. We compared our registration results with a quasigold standard based on a voxel-based affine registration using mutual information on the same datasets. The quasigold standard was generated using the approach in [1]; here the liver is first segmented by a radiologist, and the segmented images are then registered. Intrahepatic landmarks positioned at vessel bifurcations by an experienced radiologist in both datasets were used to compare registration accuracy by measuring dislocation of the landmarks after registration.

## 4. Results

### 4.1. Segmentation of Kidney

 First, we evaluated the performance of our method in the segmentation of the kidney. The kidney moves several centimeters during breathing but is, in a first approximation, a rigid organ.  Table 1 presents Dice similarity coefficients, averaged contour misalignment, and Hausdorff distances in relation to the gold standard as well as intraregion uniformity and gray level contrast. Our method showed subvoxel accuracy with a mean averaged contour misalignment of 0.504 mm (Table 1), which is below the the voxel size after subsampling of over 1.00 mm. 

Furthermore, the averaged Hausdorff distance of 3.505 mm (see Table 1) is quite acceptable. To show this, we compared the results achieved with our method with those of manual segmentation by experts in Table 2, where we used the same gold standard to measure the Dice similarity coefficient, averaged contour misalignment, and Hausdorff distance. Our method has a slightly higher misalignment of 4.482 mm compared with the experts average of 4.181 mm. However, this value is well within the subvoxel range. 

To test the goodness features, which are independent of the gold standard, we compared the quality of our segmentation method directly with that of experts. The results are presented in Table 3. The paired *t*-test was used to investigate for significant differences. With a *P* value of 0.2466, we found no significant difference in intraregion uniformity. Performance of the program was significantly better (*P* = 0.0028) using signal contrast for evaluation. 

### 4.2. Registration of Kidney

**Figure 1:**
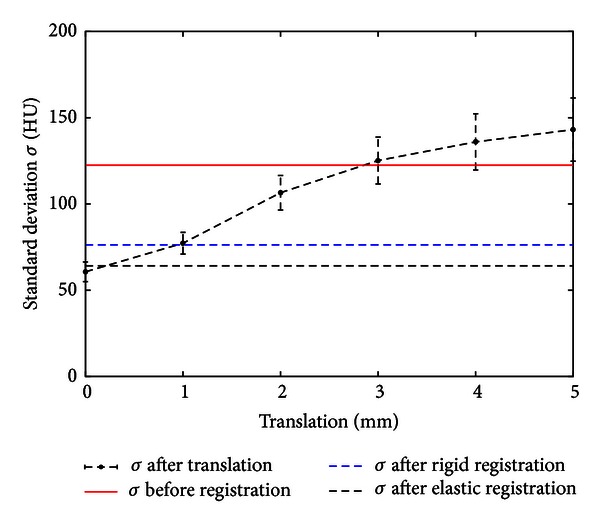
Comparison of rigid registration versus elastic registration (error bars: standard error).

 To compare our registration method with affine registration using mutual information, the paired *t*-test was used to estimate the level of reduction of standard deviation of image intensity after affine and elastic registration. As can be seen from Table 4 our method achieved a 15.11% better quality with a high confidence of 99%. This value corresponds approximately 1 mm correction of translation (see Figures 1 and 3).

### 4.3. Registration of Liver

 The liver was investigated as an example of a deformable organ. Accuracy of registration of our new method was studied using up to four landmarks per liver. Interventional liver MRI and CT examinations were performed within one hour. The paired *t*-test was used to compare landmark dislocation between both registration methods. The first column of Table 5 shows that our method has an average landmark dislocation of 4.32 mm. Affine registration yields a significantly (*P* = 0.000001) smaller dislocation of 3.26 mm. 

The difference between the two registration methods was also investigated using the Wilcoxon signed-rank test, where the correlation value between the two dislocation tests was also calculated. No significant correlation between the registration accuracy of both methods was found, with landmarks using the Wilcoxon signed-rank test. The correlation between landmark dislocations of affine and elastic registration was found to be 0.49. Therefore, registration accuracy did not significantly depend on individual image quality. This indicates that the landmarks were well set and are very well suited for our evaluation purpose.

**Figure 2:**
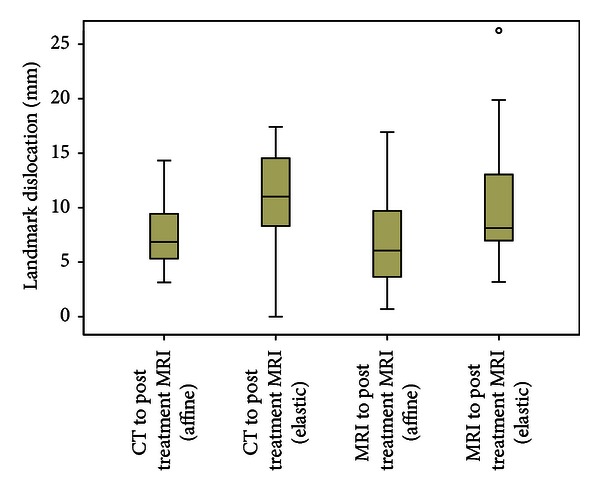
Boxplot of standard deviation of different registration methods to postinterventional MRI.

**Figure 3:**
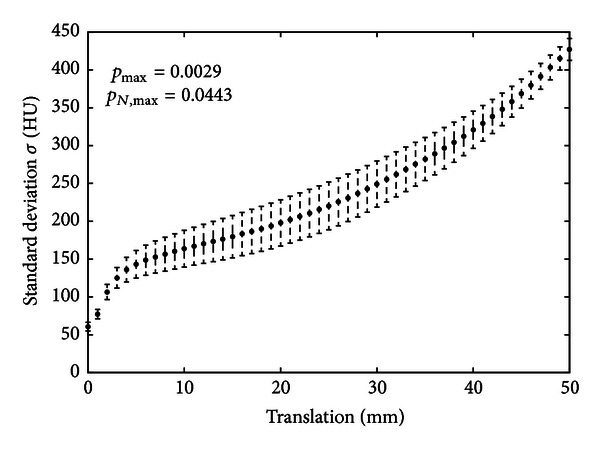
Characteristics of the standard deviation of image intensity differences after translation with the error bars demonstrating the standard error.

Furthermore, the registration methods were compared using interventional and follow-up image datasets acquired 12 weeks later. The results for coregistration of CT to follow-up MRI and interventional MRI to follow-up MRI are presented in Table 5 and in the boxplot in Figure 2, respectively. There is a clear drop in quality for both affine and elastic registrations, which is attributable to greater deformation resulting from the long interval between the two examinations. In comparison to affine registration, our method shows nearly two times greater degradation in each case. Moreover, there are also some cases in which our method did not perform better (compare *N*_*p*_ and *N*_*l*_ in Table 5). 

## 5. Discussion

 This paper presents a novel approach of elastic registration based on the coregistration of surface and volume interpolation. An important feature of our approach is that volume deformation is interpolated solely from geometric changes of the surface. We validated our approach by comparison with affine registration as the gold standard. We performed a series of tests advancing from rigid objects (the kidney), to predominantly affinely deformed objects (liver with interventional images), to elastically deformed objects (liver with follow-up images). In the last test, the liver was definitely strongly deformed due to the localized effects of brachytherapy irradiation.

Applied to the kidney, our registration method significantly improves movement correction compared with affine registration. The kidney is a relatively rigid organ and there is relatively little deformation in the 4D CT time series due to short duration of image acquisition. The result indicates that our elastic registration method performs well based on effective first correction of affine registration and can adequately correct for displacement of rigid organs such as the kidney when there is relatively little deformation inside the organ.

In contrast, for multimodal elastic registration of the liver, which is more elastic than the kidney and was deformed by irradiation in our tests, our method showed poorer performance in terms of quality compared with the twelve-parameter affine registration method. Landmark dislocation determined with affine registration seems to be a quasigold standard, which yielded 1.06 mm dislocation for quasi-simultaneously acquired interventional MRI and CT, 5.40 mm for interventional CT and a follow-up MRI, and 4.14 mm for interventional MRI and follow-up MRI. The poorer image quality of interventional MRI (signal-to-noise ratio, signal homogeneity, and spatial resolution) reduces registration accuracy for affine registration and especially for elastic registration. In principle, elastic registration should improve matching of a liver deformed by radiation treatment. The elastic registration method applied in the present study uses only information on the liver surface. In contrast, the affine registration method uses the image information of the entire liver, thus yielding a more accurate registration result. A technique using additional internal information beneath the surface might yield better results [10].

A limitation of the present study as well as of most studies using real medical images is the reliability of the validation method. Often, investigators use artificial image data for validation of their registration method, failing to take real registration problems into account. Several approaches have been proposed to validate registration accuracy; for example, Schnabel et al. validated their elastic registration method using a biomechanical model [36]. In the appendix to this paper, we demonstrate that the standard deviation in an ROI might also be a measure for comparing different coregistration methods, for example, comparison of affine registration with elastic registration as applied in the current study or by Rueckert et al. [13]. Registration accuracy can be estimated absolutely by comparing the discrepancy of the actual registration with that of landmarks defined by experts. To reduce further intra- and interexpert variability, we additionally applied an iterative expectation maximization method using multiple segmentations [33, 34].

## 6. Conclusions

 Existing methods of image registration yield unreliable results when applied to register an organ, such as the liver, that has been transformed, for example, by treatment. A more sophisticated method would be useful in this case. We have tested an approach based on coregistration of organ surfaces and interpolation of internal space. The technique has been shown to work when applied to rigid organs. However, the method was developed for application to serial datasets acquired to monitor the outcome of treatment. Our data show that the method yields unsatisfactory results when used to register an organ, such as the liver, that has been transformed by treatment, for example, radiation therapy.

Obviously, taking into account treatment-related changes on the surface is not sufficient to determine internal changes of the liver. Thus, to monitor radiation therapy of the liver, we need an elastic registration approach that uses surface information as well as information on internal organ structures in order to yield better results than 12-parameter affine registration.

## Figures and Tables

**Table 1:** Segmentation quality.

Image	DSC	$\overset{-}{d}$ (mm)	*d*_*H*_ (mm)	*U *	GC
t06 (P1)	0.958	0.575	4.482	0.927	0.311
t07 (P1)	0.966	0.450	3.153	0.928	0.311
t09 (P1)	0.971	0.351	2.104	0.921	0.248
t08 (P2)	0.960	0.628	4.275	0.910	0.144
t16 (P2)	0.966	0.517	3.512	0.918	0.151

Mean	0.964	0.504	3.505	0.921	0.233

DSC: dice similarity coefficient. $\overset{-}{d}$: averaged contour misalignment. *d*_*H*_: Hausdorff distance. *U*: intraregion uniformity. GC: gray level contrast.

**Table 2:** Comparison of segmentation quality between experts (averaged) and our program.

Expert	DSC	$\overset{-}{d}$ (mm)	*d*_*H*_ (mm)	*U *	GC
1	0.977	0.297	3.590	0.927	0.307
2	0.978	0.358	2.816	0.926	0.303
3	0.970	0.431	6.084	0.926	0.308
4	0.966	0.556	3.862	0.927	0.313
5	0.963	0.546	4.555	0.926	0.307

Mean	0.971	0.438	4.181	0.927	0.308

Program	0.958	0.575	4.482	0.927	0.311

DSC: dice similarity coefficient. $\overset{-}{d}$: averaged contour misalignment. *d*_*H*_: Hausdorff distance. *U*: intraregion uniformity. GC: signal contrast.

**Table 3:** Comparison of intraregion uniformity and signal contrast between program and gold standards.

Image	*U*_*g*_	*U*_prg_	GC_*g*_	GC_prg_
t06 (P1)	0.9269	0.9265	0.3089	0.3108
t07 (P1)	0.9280	0.9278	0.3094	0.3109
t09 (P1)	0.9214	0.9214	0.2446	0.2475
t08 (P2)	0.9099	0.9099	0.1419	0.1443
t16 (P2)	0.9175	0.9176	0.1472	0.1508

Mean	0.9207	0.9206	0.2304	0.2329

*P *	0.2466		0.0028	

*U*_*g*_: intraregion uniformity of gold standard. *U*_prg_: intraregion uniformity of program. GC_*g*_: signal contrast of gold standard. GC_prg_: signal contrast of program. *P*: two-sided level of significance of the *t*-test for *U*_prg_ versus *U*_*g*_ and GC_prg_ versus GC_*g*_.

**Table 4:** Comparison: rigid registration versus elastic registration.

Image pair	*σ*_*v*_	*σ*_*s*_	*σ*_*e*_
t07 → t09 (P1)	119.11	70.97	58.33
t20 → t05 (P1)	93.47	77.89	52.26
t12 → t20 (P1)	123.06	58.61	43.91
t09 → t13 (P2)	130.52	78.83	75.99
t07 → t05 (P2)	121.13	64.20	61.66
t11 → t08 (P2)	152.16	97.06	92.26
t16 → t01 (P2)	117.60	86.95	64.93

$\overset{-}{\sigma }$	122.43	76.36	64.19

Reduction of $\overset{-}{\sigma }$		37.63%	47.57%

*P *		0.01	

*σ*_*v*_: standard deviation before registration. *σ*_*s*_: standard deviation after rigid registration. *σ*_*e*_: standard deviation after elastic registration. *P*: two-sided significance of *t*-test for *σ*_*s*_ versus *σ*_*v*_ and *σ*_*e*_ versus *σ*_*v*_.

**Table 5:** Comparison of landmark dislocation with different registration methods.

Registration	CT to iMRI	CT to pMRI	iMRI to pMRI
*N*_*p*,*A*_	20	20	20
*N*_*l*,*A*_	76	76	77
${\overset{-}{d}}_{A}$ (mm)	3.26	6.58	6.58
*σ*_*A*_	1.25	3.31	3.25
*N*_*p*,*E*_	20	15	14
*N*_*l*,*E*_	76	56	53
${\overset{-}{d}}_{E}$ (mm)	4.32	11.98	10.72
*σ*_*E*_	1.94	5.62	5.60
*P*	0.000001	*≈*0	*≈*0

(iMRI: intrainterventional MRI, pMRI postinterventional MRI.) N_p,  A_: number of patients (affine registration). N_l,A_: number of landmarks (affine registration). ${\overset{-}{d}}_{A}$: averaged dislocation (affine registration). σ_A_: standard deviation (affine registration). N_p,E_: number of patients (elastic registration). N_l,E_: number of landmarks (elastic registration). ${\overset{-}{d}}_{E}$: averaged dislocation (elastic registration). σ_E_: standard deviation (elastic registration). *P*: two-sided level of significance of *t*-test for d_A_ versus d_E_ in the same column.

## Appendix

### A. Standard Deviation of Image Intensity as Goodness Feature

#### A.1. Theory

We assume that the change in intensity is small in the region of interest (ROI) but large between the ROI and its background. Therefore, a more precise registration method should yield a smaller variance of intensity and poorer registration results when there is significantly larger variance in the image signal difference inside the ROI of the registered image. The standard deviation of the signal intensities of the reference and the coregistrated image can be used as a measure.

#### A.2. Methods of Experimental Verification

The assumption was validated using two kidneys segmented by experts. The standard deviation of intensity between image intensities was determined after translation of the kidney image. We used five images from *P*.#1 and four from *P*.#2. The measurement was taken after every expanding translation with a step length of one voxel (1 mm). The greatest translation appropriated two times the maximum movement of the kidney that was estimated using surface registration between the kidneys. Each measurement was repeated 3000 times using randomly chosen translations in varying directions. For each kidney the average over all repetitions of each translation step was used. Finally, the average signal difference overall kidneys was calculated. In order to show the significance of the changes we further applied two different paired *t*-tests: the first one compared the changes between each step and zero translation and the second compared between neighboring steps.

#### A.3. Results of Experimental Verification

The dependence of the signal intensity difference on the dislocation between reference and coregistrated image is demonstrated in Figure 3. The large number of repetitions yielded maximum standard error of 1.68%, represented by the error bar. A continuous increase in the standard deviation of the image difference with dislocation is demonstrated. Maximum significance between neighboring steps is *p*_*N*,max⁡_ = 0.0443; maximum significance against zero translation is *p*_max⁡_ = 0.0029. Thus, the reduction in standard deviation after each translation step of one voxel can be interpreted as a quality degradation of registration.
